# Protective Effects of Luteolin on *Glaesserella parasuis*-Induced Injury: An In Vitro Study with Porcine Vascular Endothelial Cells

**DOI:** 10.3390/antibiotics14080824

**Published:** 2025-08-12

**Authors:** Pu Guo, Xuwen Liu, Xiaoyi Li, Awais Ihsan, Zhongyuan Wu, Shulin Fu, Chun Ye, Yinsheng Qiu, Xu Wang, Qirong Lu, Yu Liu

**Affiliations:** 1 Hubei Key Laboratory of Animal Nutrition and Feed Science, Wuhan Polytechnic University, Wuhan 430023, China; guopu@whpu.edu.cn (P.G.); liuxuwen548936@126.com (X.L.); 18051080269@163.com (X.L.); zhongywu@whpu.edu.cn (Z.W.); shulinfu@whpu.edu.cn (S.F.); yechun@whpu.edu.cn (C.Y.); qiuyinsheng6405@whpu.edu.cn (Y.Q.); qirongluvet@whpu.edu.cn (Q.L.); 2 Wuhan Engineering and Technology Research Center of Animal Disease-Resistant Nutrition, School of Animal Science and Nutritional Engineering, Wuhan Polytechnic University, Wuhan 430023, China; 3 Department of Biosciences, COMSATS University Islamabad (CUI), Islamabad 45550, Pakistan; awais@cuisahiwal.edu.pk; 4 National Reference Laboratory of Veterinary Drug Residues (HZAU) and MAO Key Laboratory for Detection of Veterinary Drug Residues, Huazhong Agricultural University, Wuhan 430070, China; wangxu@mail.hzau.edu.cn

**Keywords:** *Glaesserella parasuis*, luteolin, vascular endothelial permeability, CD44, infection

## Abstract

**Background:** *Glaesserella parasuis* (GPS) is a conditional pathogen that colonizes the upper respiratory tract in pigs and causes Glässer’s disease, resulting in high morbidity and mortality in piglets. GPS infection increases the vascular endothelial permeability, but the mechanism has not been fully elucidated. Luteolin (Lut) is a naturally occurring flavonoid found in plants such as vegetables, herbs, and fruits, but its potential to treat the increased vascular endothelial permeability caused by GPS infection has not been evaluated. **Results:** This study revealed that GPS infection induces increased vascular endothelial permeability in porcine iliac artery endothelial cells (PIECs) by increasing the gene expressions of tumor necrosis factor (TNF), interleukin 6 (IL-6), IL-8, and IL-1β, and by regulating F-actin cytoskeleton reorganization. Mechanistically, GPS infection or Cluster of differentiation 44 (CD44) overexpression significantly increased the expressions of vascular-endothelial-permeability-related proteins (CD44; vascular endothelial growth factor (VEGFA); matrixmetalloProteinase-3 (MMP-3); MMP-9; and SRC proto-oncogene, non-receptor tyrosine kinase (c-Src)) and increased the vascular endothelial permeability; these changes were alleviated by a Lut treatment or CD44 silencing in the PIECs. **Conclusions:** This study comprehensively illustrates the potential targets and molecular mechanism of Lut in alleviating the GPS-induced increase in vascular endothelial permeability. The CD44 pathway and Lut may be an effective target and antibiotic alternative, respectively, to prevent the increased vascular endothelial permeability caused by GPS.

## 1. Introduction

*Glaesserella parasuis* (GPS) is a conditional pathogen that colonizes the upper respiratory tract in pigs and causes Glässer’s disease, resulting in high morbidity and mortality in piglets [1]. Glässer’s disease is typically characterized by fibrinous polyserositis, arthritis, meningitis, and frequent symptoms of pneumonia. Severe infection causes sepsis and death in pigs and leads to significant economic losses to the swine industry worldwide [2,3]. From 2005 to 2019, the overall prevalence rate of GPS infection in pigs in China was 27.8%, with the highest prevalence rate of 41% from 2011 to 2015 [4]. On some pig farms, the mortality rate of pigs infected with GPS can reach 50% [4]. Glässer’s disease is also associated with vascular damage and vascular inflammation in pigs [5]. In most organs, vascular endothelial cells form a dynamic barrier between blood and tissues [6], and they are an unusual target of bacterial infection [7]. Notably, endothelial hyperpermeability has been identified as a marker of the pathophysiology of acute and chronic inflammatory diseases, such as sepsis [8]. However, there has been insufficient research on the increased vascular endothelial permeability caused by GPS infection. Therefore, elucidating the molecular mechanism of increased vascular endothelial permeability induced by GPS infection and developing adjuvant therapies or effective drugs to control this phenomenon have important scientific significance and therapeutic value for reducing the risk of GPS infection.

Cluster of differentiation 44 (CD44) is an adhesion molecule expressed by various cell types, including endothelial cells, which is critical for maintaining vascular endothelial permeability [9,10]. Plasma levels of soluble CD44 are significantly elevated in mice subjected to a sham operation or cecal ligation and puncture-induced sepsis; the ectodomain of CD44 dose-dependently increases the endothelial barrier permeability in human umbilical vein endothelial cell (HUVEC) monolayers [11]. Compared with those in CD44 wild-type mice, the vascular endothelial permeability is increased and the secretion of inflammatory factors induced by staphylococcal enterotoxin B exposure are significantly reduced [12]. Moreover, in CD44 knockout mice, the protective effect of high-molecular-weight hyaluronic acid on lipopolysaccharide (LPS)-induced pulmonary vascular hyperpermeability is significantly reduced [13]. The above studies indicate that CD44 is a regulator of vascular endothelial permeability, although whether CD44 is also involved in the vascular endothelial permeability injury induced by GPS infection has not yet been reported.

Luteolin (Lut) is a naturally occurring flavonoid found in plants such as vegetables, herbs, and fruits. It is considered to have health-promoting properties as part of the human diet and has been reported to have a wide range of pharmacological activities [14]; the ability to regulate vascular endothelial permeability [15]; anti-inflammatory [16], antioxidant [17], anticancer [18], and neuroprotective [19] effects; and the ability to ameliorate intestinal injury [20]; among other functions. Moreover, Lut can reduce the endothelial permeability and apoptosis induced by hypoxia-reoxygenation injury by regulating the Wnt/β-catenin pathway [15]. In a human blood-brain barrier model, Lut could protect the barrier function after exposure to fibrillary Aβ by preserving transendothelial resistance and reducing aggravated endothelial permeability [21]. Lut also prevents LPS-induced inflammatory responses, including increased pulmonary vascular permeability and tumor necrosis factor (TNF) and interleukin 6 (IL-6) production in mice [22]. The above studies indicate that Lut has a strong ability to counteract vascular dysfunction, but whether it can ameliorate the aggravated vascular endothelial permeability caused by GPS infection is unclear.

The present study explored the increased vascular endothelial permeability caused by GPS infection, and the potential mechanism by which Lut could counteract this phenomenon via the CD44 signaling pathway in porcine iliac artery endothelial cells (PIECs). The aim of this study was to determine whether Lut can be considered a promising natural agent to alleviate increased vascular endothelial permeability in GPS-infected piglets.

## 2. Results

### 2.1. Dose-Dependent Cytotoxicity of Lut on PIECs

**Figure 1:**
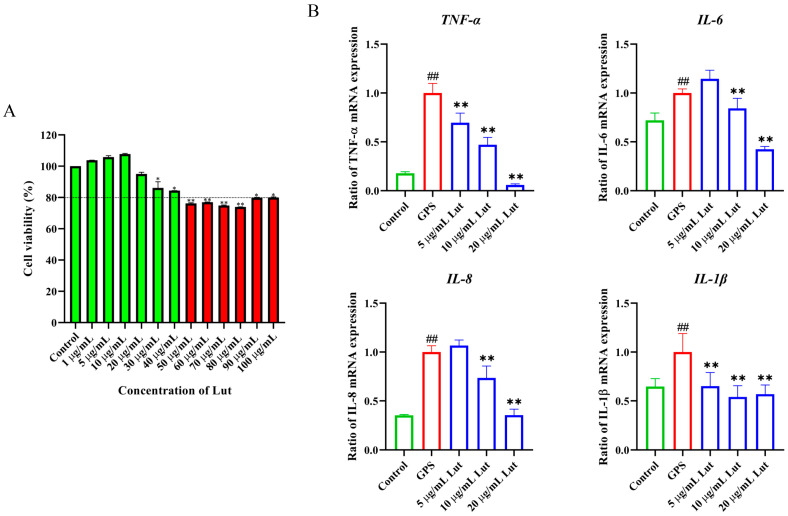
Lut-modulated GPS-induced increase in the expression of inflammatory genes in the PIECs. (**A**) The cytotoxicity of Lut on the PIECs was determined via a CCK8 kit. The PIECs were treated with Lut (1–100 μg/mL) for 12 h. (**B**) RT-qPCR results showing that Lut alleviated the GPS-induced expression of inflammatory genes in the PIECs. The PIECs were pretreated with Lut for 1 h and then cocultured with GPS for another 12 h. ## *p* < 0.01 for the control group vs. the GPS group; * *p* < 0.05 for the Lut group vs. the GPS group for (**B**) (or control group for (**A**)), ** *p* < 0.01 for the Lut group vs. the GPS group for (**B**) (or control group for (**A**)).

The viability of PIECs treated with different concentrations of Lut was evaluated with a CCK8 kit. Lut exerted dose-dependent effects on the PIECs. When the Lut concentration exceeded 40 μg/mL, the cell survival rate was <80%. In other words, a concentration of >40 μg/mL resulted in significant cytotoxicity (*p* < 0.01) (Figure 1A). Therefore, the Lut concentration for the subsequent experiments was <40 μg/mL.

### 2.2. GPS Increased Vascular Endothelial Permeability in PIECs

**Figure 2:**
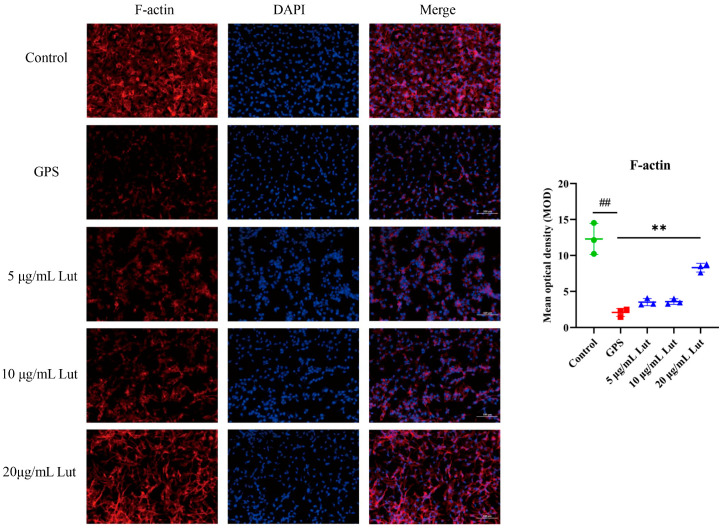
Lut reversed the F-actin cytoskeletal reorganization induced by GPS. The PIECs were pretreated with Lut for 1 h and then cocultured with GPS for another 12 h. The intensity of F-actin staining was quantified via ImageJ software (https://imagej.net/ij/download.html). ## *p* < 0.01 for the control group vs. the GPS group; ** *p* < 0.01 for the Lut group vs. the GPS group.

To establish a vascular endothelial permeability model, PIECs were infected with GPS, the expression of inflammatory genes was determined, and filamentous actin (F-actin) was labeled with rhodamine phalloidin. Compared with those in the control group, the TNF, IL-6, interleukin 8 (IL-8), and interleukin-1beta (IL-1β) gene expressions were significantly increased in the GPS-infected group (Figure 1B). Moreover, compared with that in the control group, the intensity of F-actin staining in the interior of the cells in the GPS-infected group was significantly reduced (Figure 2), indicating that GPS damaged the cytoskeletal structure of the cytoskeleton and increased the vascular endothelial permeability. These findings suggest that GPS infection of PIECs can induce inflammatory injury to vascular endothelial cells and increase vascular endothelial permeability.

### 2.3. GPS Increased the Expression of Vascular-Endothelial-Permeability-Related Proteins in PIECs

**Figure 3:**
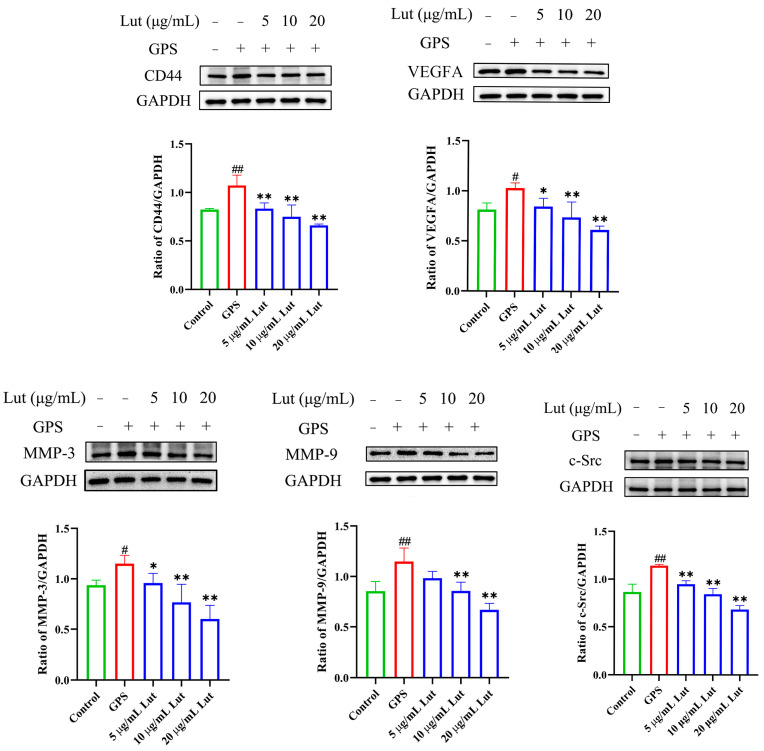
Lut counteracted the increase in vascular endothelial permeability-related proteins expression induced caused by GPS. Lut reduced the expression of vascular-endothelial-permeability-related proteins induced by GPS in the PIECs, including CD44, VEGFA, MMP-3, MMP-9, and c-Src induced by GPS treated in the PIECs. ImageJ software was used for the densitometry. # *p* < 0.05 and ## *p* < 0.01 for the control group vs. the GPS group; * *p* < 0.05 and ** *p* < 0.01 for the control group vs. the GPS group.

The expression of vascular-endothelial-permeability-related proteins was measured in the PIECs after GPS infection to evaluate the increased vascular endothelial permeability. Western blotting revealed significantly increased VEGFA, MMP-3, CD44, MMP-9, and c-Src protein expressions in the GPS-infected group compared with the control group (Figure 3). The above studies indicate that GPS increases the expression of proteins related to vascular endothelial permeability in PIECs.

### 2.4. Lut Attenuated the GPS-Induced Increase in Vascular Endothelial Permeability by Suppressing the CD44 Pathway in PIECs

**Figure 4:**
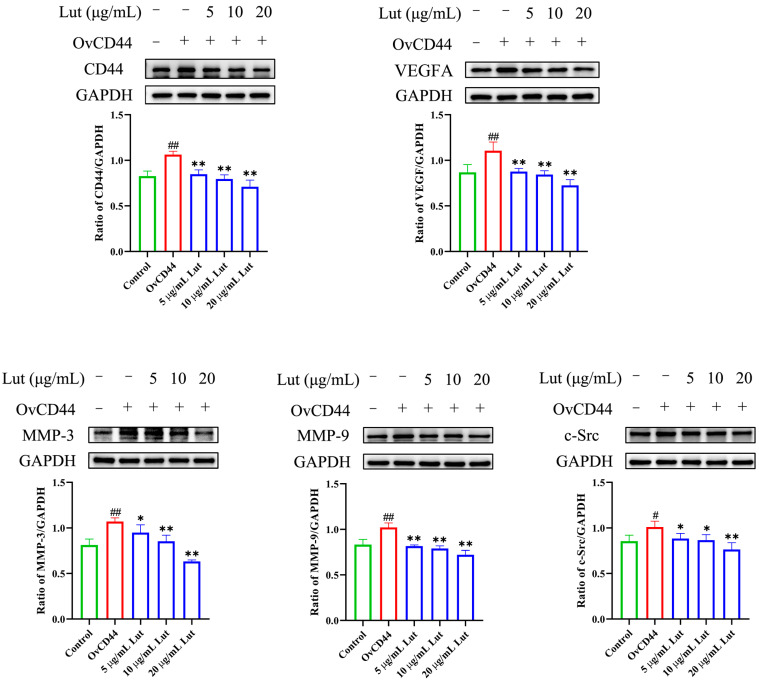
Lut alleviated the CD44 overexpression–induced increased expression of vascular endothelial permeability-related proteins in PIECs. Pretreatment with Lut alleviated the CD44-overexpression-induced upregulation of vascular-endothelial-permeability-related proteins, including CD44, VEGFA, MMP-3, MMP-9, and c-Src, in the PIECs. ImageJ software was used for densitometry. OvCD44 indicates CD44 overexpression. # *p* < 0.05 and ## *p* < 0.01 for the control group vs. the OvCD44 group; * *p* < 0.05 and ** *p* < 0.01 for the control group vs. the OvCD44 group.

**Figure 5:**
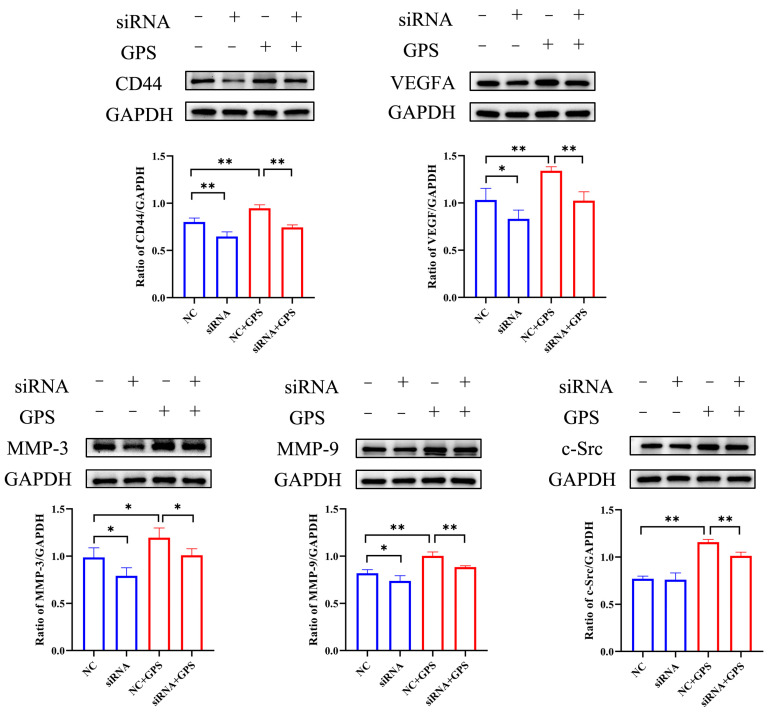
Silencing CD44 reduced the GPS–induced increase in the expression of vascular endothelial permeability-related proteins in PIECs. Silencing CD44 decreased the GPS-induced upregulation of vascular-endothelial-permeability-related proteins, including CD44, VEGFA, MMP-3, MMP-9, and c-Src, in the PIECs. ImageJ software was used for the densitometry. siRNA indicates the silencing of CD44. NC indicates negative control siRNA. * *p* < 0.05; ** *p* < 0.01.

To evaluate whether Lut could reduce the increased permeability of vascular endothelial cells induced by GPS and its mechanism, a comprehensive evaluation was performed by combining GPS, a Lut treatment, and CD44 overexpression (OvCD44) and CD44 interference assays. The results showed that 5, 10, or 20 μg/mL Lut significantly decreased the TNF, IL-6, IL-8, and IL-1β gene expressions (Figure 1B) and alleviated the F-actin cytoskeleton reorganization (Figure 2) caused by GPS. Moreover, 5, 10, or 20 μg/mL Lut significantly decreased the increased expression of vascular-endothelial-permeability-related proteins (CD44, VEGFA, MMP-3, MMP-9, and c-Src) induced by GPS to varying degrees (Figure 3). OvCD44 and CD44 interference assays were used to simulate GPS and Lut treatments, respectively. Compared with those in the control group, the CD44, VEGFA, MMP-3, MMP-9, and c-Src protein expressions were significantly increased in the OvCD44 group (Figure 4). However, after the Lut treatment, compared with those in the OvCD44 group, the levels of vascular-endothelial-permeability-related proteins (CD44, VEGFA, MMP-3, MMP-9, c-Src) were significantly decreased to varying degrees in the Lut treatment groups (Figure 4). Moreover, the CD44, VEGFA, c-Src, MMP-9, and MMP-3 protein expressions were significantly lower in the CD44 siRNA + GPS group than in the negative control siRNA (NC) + GPS group (Figure 5). These findings indicate that Lut can attenuate the GPS-induced increase in vascular endothelial permeability by suppressing the CD44 pathway in the PIECs.

## 3. Discussion

GPS is a conditional pathogen that colonizes the upper respiratory tract in pigs and causes Glässer’s disease, resulting in high morbidity and mortality in piglets [1]. The characteristic lesion of Glässer’s disease is fibrinous inflammation. GPS has a strong ability to adhere to and invade porcine aortic endothelial cells [23], suggesting that GPS infection can cause vascular endothelial dysfunction and a systemic inflammatory response. The preservation of vascular endothelial cell barrier integrity is essential for normal vascular homeostasis [13], and barrier dysfunction is closely related to the dysregulation of vascular endothelial permeability [8]. Therefore, exploring the mechanism of increased vascular endothelial permeability caused by GPS infection and finding effective adjuvant therapies or agents to control vascular endothelial permeability to reduce the risk of GPS infection are highly important. In the present study, GPS-infected PIECs were used to establish a model of increased vascular endothelial permeability. Lut alleviated the increase in vascular endothelial permeability during GPS infection by inhibiting the CD44 signaling pathway. These findings suggest that Lut and CD44 represent a candidate agent and candidate target, respectively, for alleviating the increased vascular endothelial permeability induced by GPS.

Increased vascular endothelial permeability is closely related to dysregulation of inflammatory factors and F-actin cytoskeleton reorganization. In rats, intravenous tail injection of TNF for 24 h can increase lung vascular permeability [24]. Treatment of HUVECs with TNF altered F-actin stress fibers and significantly increased endothelial permeability [25,26]. IL-8 stimulation can induce F-actin reorganization and increase vascular endothelial permeability, and the inhibition of Src could abolish IL-8-induced permeability in primary human microvascular endothelial cells [27]. MC-LR increased the vascular permeability of HUVECs via IL-8/CXCR2 signaling [28]. Moreover, IL-1β induced a dose-dependent increase in endothelial permeability by activating the Src kinase pathway in porcine retinal endothelial cells [29]. In addition, IL-6 or IL-6/IL-6R stimulation significantly increases the vascular endothelial permeability of human renal glomerular endothelial cells by downregulating VE-cadherin expression [30]. IL-6 stimulation caused F-actin cytoskeleton reorganization and increased permeability in human pulmonary artery endothelial cells [31]. IL-6 can increase the vascular endothelial permeability by rearranging F-actin filaments and changing the shape of endothelial cells in bovine vascular endothelial cells [32]. The abovementioned studies demonstrated that dysregulation of inflammatory factors and F-actin cytoskeleton reorganization are critical markers of increased vascular endothelial permeability. Consistent with the findings of the present study, GPS promoted the expression of inflammatory genes (TNF, IL-6, IL-8, and IL-1β) and mediated F-actin cytoskeleton reorganization, indicating that GPS can increase the vascular endothelial permeability. Treatment with Lut alleviated the increased vascular endothelial permeability induced by GPS.

CD44 is a widely distributed cell surface glycoprotein that is critical for maintaining vascular endothelial permeability [9,10]. Compared with those in CD44 wild-type mice, the increased vascular endothelial permeability and the secretion of inflammatory factors induced by staphylococcal enterotoxin B exposure are markedly reduced in CD44-deficient mice [12]. Plasma levels of soluble CD44 are significantly elevated in mice subjected to a sham operation or cecal ligation and puncture-induced sepsis, and the ectodomain of CD44 dose dependently increases endothelial barrier permeability in HUVEC monolayers [11]. However, compared with C57BL/6 wild-type mice, CD44-deficient mice show significant increases in proinflammatory cytokines and a more permeable cerebral vasculature [33]. These studies indicate that under normal physiological conditions, CD44 is a necessary factor for maintaining vascular endothelial permeability. Under external stimuli or infection, excessive CD44 increases vascular endothelial permeability and induces the secretion of inflammatory factors. In the present study, GPS of PIECs significantly increased the CD44 expression and vascular endothelial permeability. This activation was significantly inhibited by Lut, suggesting that CD44 silencing and Lut treatment may play important roles in alleviating the increased vascular endothelial permeability induced by GPS.

VEGFA, MMP-3, MMP-9, and c-Src play important roles in regulating vascular endothelial permeability. VEGFA significantly increased vascular endothelial permeability in mice or in primary murine lung endothelial cells [34], and induced lung vessel permeability during acute lung injury [35]. MMP-3 stimulation can increase the vascular permeability in brain microvascular endothelial cells and in wild-type + MMP 3 mouse brains [36]. In a study with HUVECs, compared with the control group, the MMP-9-treated group showed significantly increased endothelial permeability [37]. Hydrogen sulfide reduces the increase in blood-brain barrier permeability induced by hyperhomocysteinemia by inhibiting MMP-9 [38]. c-Src depletion significantly attenuates paraquat-induced cell toxicity and microvascular endothelial hyperpermeability in endothelial monolayers [39]. eNOS induces vascular hyperpermeability through the VEGFA/VEGFR2/c-Src/VE-cadherin pathway [40]. Consistently, in the present study, GPS or CD44 overexpression significantly increased the expression of vascular-endothelial-permeability-related proteins (CD44, VEGFA, MMP-3, MMP-9, and c-Src) and increased the vascular endothelial permeability, which could be alleviated by Lut treatment or CD44 silencing. Hence, inhibition of the CD44 pathway can alleviate the enhanced vascular endothelial permeability caused by GPS.

Although the present study provides novel insights into the increased vascular endothelial permeability caused by GPS, several limitations remain. First, whether Lut can target CD44 to regulate increased vascular endothelial permeability remains to be determined. Second, the in-depth mechanism by which CD44 regulates the expression of VEGFA, MMP-3, MMP-9, and c-Src remains to be explored. Third, the potential of Lut to ameliorate GPS-induced increases in vascular endothelial permeability needs to be evaluated in animal models.

## 4. Materials and Methods

### 4.1. Reagents and Chemicals

Lut (CAS No. 491-70-3) was purchased from Aladdin (Shanghai, China). Dimethyl sulfoxide (DMSO) (CAS No. 67-68-5) was purchased from Yeasen (Shanghai, China).

### 4.2. Cell and Bacterial Culture

PIECs were gifted by Professors Hongkui Wei and Menghong Dai from Huazhong Agricultural University. The cells were cultured in RPMI medium containing 10% fetal bovine serum (PAN, Aidenbach, Germany) and 1% penicillin–streptomycin (Gibco, Billings, MT, USA) in an incubator at 37 °C with 5% CO_2_.

The GPS strain SH0165 serovar 5 was cultured in tryptic soy broth (Hopebio, Jinan, China) containing 10% newborn calf serum (Tianhang, Zhengzhou, China) and 1% nicotinamide adenine dinucleotide (Sigma, St. Louis, MO, USA) in an incubator at 37 °C.

The bacterial infection time and methods were as described previously [2]. The PIECs (1 × 10^5^) were seeded into 24-well plates (Costar, Washington, DC, USA) and pretreated with different concentrations Lut for 1 h. Next, GPS (1 × 10^6^ CFU/mL) were added and co-cultured for 12 h at 37 °C with 5% CO_2_.

### 4.3. Cell Viability Assay

The PIECs were seeded in 96-well plates and then incubated with different concentrations of Lut (1–100 μg/mL) for 12 h. After the treatment, the percentage of cell survival was calculated according to the instructions of the cell counting kit-8 (CCK8) kit (Vazyme, Nanjing, China), which is based on the intracellular dehydrogenase activity of viable cells, which is indirectly reflected by the amount of water-soluble tetrazolium salt (WST-8) reduced to water-soluble formazan.

### 4.4. RNA Isolation and Reverse Transcription Quantitative Polymerase Chain Reaction (RT-qPCR)

The PIECs were pretreated with Lut for 1 h, and then cocultured with GPS for another 12 h. RT-qPCR was carried out with ChamQ SYBR Color qPCR Master Mix (Vazyme, China) according to the manufacturer’s instructions. The 2^−ΔΔCt^ method was used to calculate the relative mRNA expression, and glyceraldehyde-3-phosphate dehydrogenase (GAPDH) was used for normalization. All primers used are listed in Table 1.

### 4.5. Western Blot

The samples were separated via 10% SDS-PAGE (Sangon Biotech, Shanghai, China) and then transferred to PVDF membranes via a protein transfer system. The membrane was incubated with 5% nonfat milk and incubated with primary antibodies (CD44 antibody (Proteintech Group, Wuhan, China) and vascular endothelial growth factor (VEGFA); MatrixmetalloProteinase-3 (MMP-3); MMP-9; and SRC proto-oncogene, non-receptor tyrosine kinase (c-Src) antibodies (all from ABclonal, Wuhan, China). Then, the membrane was incubated with secondary antibody (ABclonal, China). Finally, the protein bands were visualized with an enhanced chemiluminescence (ECL) kit (ABclonal, China) and the FluorChem E device (ProteinSimple, Shanghai, China). ImageJ software (National Institutes of Health, Bethesda, MD, USA) was used for the densitometry.

### 4.6. Immunofluorescence

The PIECs were seeded in 6-well plates containing cell slides (WHB Scientific, Shanghai, China). For F-actin staining, the cell slides were incubated with Phalloidin-iFluor 594 reagent (Abcam, Cambridge, UK) in the dark for 2 h at 37 °C and then incubated with DAPI (Beyotime, Shanghai, China) in the dark for 5 min to stain the nuclei. Images were captured via a laser-scanning confocal microscope (FV10i, Olympus, Tokyo, Japan) and analyzed via ImageJ software.

### 4.7. Cell Transfection

For the CD44 overexpression, pcDNA3.1-CD44 or pcDNA3.1 was treated with Lipo8000™ transfection reagent (Beyotime). For the RNA interference (RNAi), small interfering RNA (siRNA) against CD44 or negative control siRNA (GenePharma, Shanghai, China) was treated with the Lipo8000™ transfection reagent (Beyotime).

### 4.8. Statistical Analysis

All the results are presented as the mean ± standard deviation. The data was analyzed via ANOVA with post hoc Duncan’s correction. Significant and strongly significant differences were considered at *p* < 0.05 and *p* < 0.01, respectively.

## 5. Conclusions

Overall, the results of the present study demonstrate that GPS infection of PIECs activates the CD44 signaling pathway to increase vascular endothelial permeability and that these effects can be significantly inhibited by Lut. This study has comprehensively illustrated the potential targets and molecular mechanism of Lut in alleviating the GPS-induced increase in vascular endothelial permeability. The CD44 signaling pathway and Lut may be effective targets and antibiotic alternatives, respectively, to prevent the increased vascular endothelial permeability caused by GPS.

## Figures and Tables

**Table 1:** Primer sequences for RT-qPCR analysis.

Gene	Primer	Primer Sequence (5′→3′)
GAPDH	Forward	TCGGAGTGAACGGATTTGGC
	Reverse	TGCCGTGGGTGGAATCATAC
TNF	Forward	CGCTCTTCTGCCTACTGCACTTC
	Reverse	CTGTCCCTCGGCTTTGACATT
IL-6	Forward	TGTCGAGGCTGTGCAGATTAGT
	Reverse	ATCCACTCGTTCTGTGACTGC
IL-8	Forward	ACAGCAGTAACAACAACAAG
	Reverse	GACCAGCACAGGAATGAG
IL-1β	Forward	GCTGGAGGATATAGACCCC
	Reverse	GTTGGGGTACAGGGCAGAC

Note: GAPDH: glyceraldehyde-3-phosphate dehydrogenase, IL-1β: interleukin-1 beta, IL-6: interleukin 6, IL-8: interleukin 8, TNF: tumor necrosis factor.

## Data Availability

The data presented in this study are available upon request from the corresponding author.

## Abbreviations

The following abbreviations are used in this manuscript:

c-Src	SRC proto-oncogene, non-receptor tyrosine kinase
CCK8	Cell counting kit-8
CD44	Cluster of differentiation 44
DMSO	Dimethyl sulfoxide
ECL	Enhanced chemiluminescence
F-actin	Filamentous actin
GAPDH	Glyceraldehyde-3-phosphate dehydrogenase
GPS	*Glaesserella parasuis*
HUVEC	Human umbilical vein endothelial cell
IL-1β	Interleukin-1beta
IL-6	Interleukin 6
IL-8	Interleukin 8
LPS	Lipopolysaccharide
Lut	Luteolin
MMP-3	MatrixmetalloProteinase-3
MMP-9	MatrixmetalloProteinase-9
NC	Negative control siRNA
OvCD44	CD44 overexpression
PIECs	Porcine iliac artery endothelial cells
RNAi	RNA interference
RT-qPCR	Reverse transcription quantitative polymerase chain reaction
siRNA	Small interfering RNA
TNF	Tumor necrosis factor
VEGFA	Vascular endothelial growth factor
